# Deep cutaneous phaeohyphomycosis caused by *Cladophialophora boppii*: A case report

**DOI:** 10.1016/j.mmcr.2025.100717

**Published:** 2025-07-14

**Authors:** Thuy Thanh Cavens, Janneke de Vries, Suzanne Verleisdonk-Bolhaar, Jochem B. Buil, Arienne van Marion

**Affiliations:** a Department of Pathology VieCuri, Tegelseweg 210, 5912 BL Venlo, Netherlands; b Department of Medical Microbiology Viecuri, Tegelseweg 210, 5912 BL Venlo, Netherlands; c Department of Dermatology Viecuri, Tegelseweg 210, 5912 BL Venlo, Netherlands; d Department of Medical Microbiology, Radboud University Medical Center, Geert Grooteplein Zuid 10, 6525 GA Nijmegen, Netherlands

**Keywords:** Phaeohyphomycosis, *Cladophialphora boppii*, Deep cutaneous mycosis, Plaque lesion, Dematiaceous fungi

## Abstract

*Cladophialophora boppii* is a dematiaceous fungus rarely causing cutaneous and respiratory infections. We present a case of deep cutaneous infection in a 69-year-old patient in the Netherlands with stage IV breast cancer on dexamethasone therapy. The patient exhibited a skin plaque lesion, and biopsy revealed pigmented, septate hyphae without muriform cells, consistent with a diagnosis of phaeohyphomycosis. Surgical excision and culture confirmed *C. boppii*. Afterwards the patient received a three-week course of itraconazole, which led to infection resolution. This report and review of literature underscores the diagnostic and therapeutic approaches for managing deep cutaneous infections caused by *C. boppii*.

## 1 Introduction

Phaeohyphomycosis is a fungal infection caused by dematiaceous (melanized) fungi, which contain dark pigments (melanin) in their cell walls. This condition encompasses a large and heterogeneous group of fungal pathogens. To date, causative agents from approximately 70 genera and 150 species have been implicated in phaeohyphomycosis. Commonly reported genera include *Exophiala*, *Fonsecaea*, *Phialophora*, *Cladosporium*, and *Cladophialophora*, among others [1].

*Cladophialophora boppii* (formerly *Taeniolella boppii*) was first described in Brazil in 1983 as a causative agent of cutaneous infection [2]. It is now known to cause both superficial and deep cutaneous infections, with only four case reports detailing its cutaneous manifestations to date [[2], [3], [4], [5]]. Additionally, it has been associated with toenail infection, keratitis, and pulmonary infection in an immunocompromised lung transplant recipient [[6], [7], [8]].

Isolates of *C. boppii* have been reported in several European countries, including Belgium (IHEM 20632), Germany (CBS 110028), the Netherlands (CBS 110029), Austria (CBS 316.56), and Spain (CBS 102826), and Brazil (CBS 126.86). In addition, clinical cases have been identified in Denmark, Brazil and Japan [2,4,[9], [10], [11]].

## 2 Case presentation

We present the case of a 69 year old female patient in the Netherlands with stage IV breast cancer. The patient had been receiving fulvestrant monotherapy for six months and dexamethasone 4 mg/day for 12 months. Leukocyte counts were within the normal range. She presented with a rapidly evolving plaque-like skin lesion on the trunk on day 0, clinically suspected to be either a basal cell carcinoma or an excoriation. A biopsy of the lesion was performed, and the material was submitted to the pathology department for histological evaluation.

**Fig. 1:**
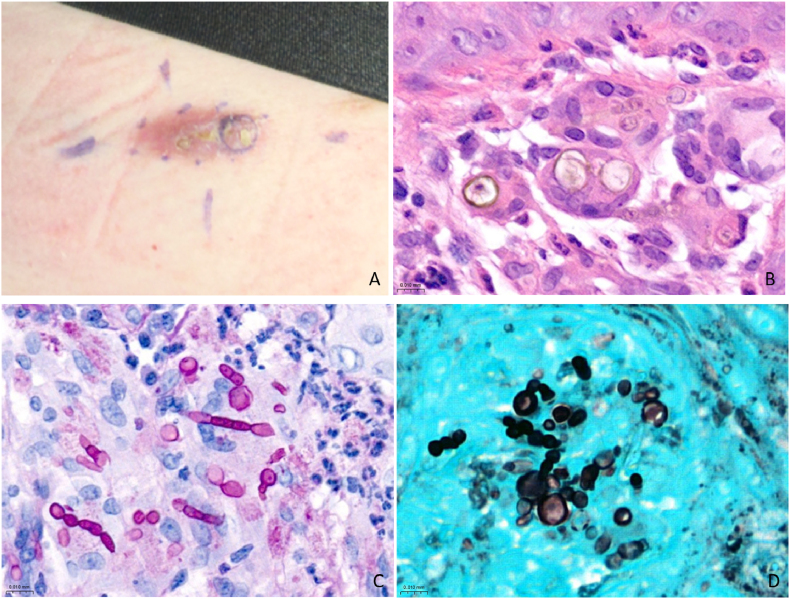
Clinical and histopathological images A) Plaque lesion with scaling on the skin of the trunk. B) Dematiaceous, pigmented yeast-like cells within multinucleated giant cells surrounded by a few neutrophils. These round fungal structures lack internal multiseptation. They are located in the papillary dermis, with epidermal keratinocytes visible at the top of the image. C) Periodic acid-Schiff diastase stain showing multiple septate hyphae, pseudohyphae and yeast-like cells with small nucleoli. The fungal structures are located in the deep dermis and surrounded by multinucleated giant cells, histiocytes and neutrophils. D) Grocott's methenamine silver stain showing multiple pseudohyphae and yeast-like cells.

Histopathological analysis revealed pseudoepitheliomatous hyperplasia with numerous neutrophilic granulocytes and multinucleated giant cells in the underlying dermis. No involvement of the subcutaneous tissue was observed. The multinucleated giant cells contained pigmented yeast-like structures, alongside predominantly pseudohyphae, and a few septate hyphae in the surrounding tissue (see Fig. 1). These findings prompted a differential diagnosis of chromoblastomycosis versus phaeohyphomycosis, both caused by dematiaceous fungi. However, the absence internal multiseptation in the yeast-like cells supported a diagnosis of phaeohyphomycosis. To further refine the diagnosis, it was confirmed that the patient had not recently traveled to tropical or subtropical regions, where chromoblastomycosis is more common.

Subsequently, the skin lesion was presumably completely excised based on clinical observation on day 7. Half of the excision was examined histopathologically, the other half was submitted to the microbiology laboratory. Cultures were initiated at the microbiology department. The samples were incubated for three weeks at 30 and 36 °C. Growth of a black fungus was detected on day 12, five days after incubation began on agar at 30 °C. The full internal transcribed spacer (ITS) region was sequenced using primers ITS1 and ITS4 [1]. Sequence analysis showed a 100 % match with the type strain of *C. boppi* (accession number: NR_131297.1), confirming *C. boppii* as the causative agent. Antifungal susceptibility testing was performed by broth microdilution following European Committee on Antimicrobial Susceptibility Testing (EUCAST) methods (Table 1).

**Table 1:** Antifungal susceptibility of *C. boppii* isolate determined using the EUCAST broth microdilution method for filamentous fungi.

Antifungal agent	MIC (mg/L) (∗)
Amphotericin B	1
Itraconazole	0.125
Voriconazole	0.25
Posaconazole	0.063
Isavuconazole	0.5
Fluconazole	>64
Miconazole	1
Olorofim	>2
5-flucytosine	4
Terbinafine	0.016
Anidulafungin	0.031
Micafungin	0.016
Fosmanogepix	0.001
Rezafungin	0.063

MIC: minimal inhibitory concentration. For anidulafungin, micafungin, fosmanogepix and rezafungin the minimal effective concentration was determined instead of the MIC.

After excision and awaiting the results of the culture, the patient started treatment with itraconazole at a dose of 200 mg/day. Due to side effects of itraconazole (severe malaise and loss of taste) and the apparently complete excision, itraconazole was discontinued after 3 weeks. During this period, no therapeutic drug monitoring (TDM) was performed. No new skin lesions were observed during the 4-month follow-up period.

## 3 Discussion

This case report underscores the rarity of *C. boppii* infections, the diagnostic challenges faced by clinicians and pathologists, and the considerations involved in treatment strategies. Our findings suggest that patients undergoing prolonged corticosteroid therapy, such as dexamethasone, may be more susceptible to deep cutaneous *C. boppii* infections. However a subcutaneous infection has also been described in an immunocompetent individual without notable predisposing factors [3].

Cutaneous phaeohyphomycosis typically presents clinically as slow-growing, solitary or multiple nodules, plaques, or papules, most commonly involving the extremities. These lesions may appear erythematous, verrucous, or ulcerative and are often misdiagnosed as basal cell carcinoma or squamous cell carcinoma [1,12]. Deep cutaneous infections caused by *C. boppii* have been reported to present as an ulcerative elevated nodule on the shin, a vegetative plaque on the foot or multiple slow-growing reddish nodules on the hands and face [[2], [3], [4], [5]].

Histopathologically, dematiaceous fungi in the skin manifest primarily as either chromoblastomycosis or phaeohyphomycosis. Phaeohyphomycosis is distinguished by the presence of pigmented septate hyphae without muriform cells. In contrast, muriform cells—also known as Medlar bodies, sclerotic cells, or "copper pennies"—are thick-walled, multiseptated structures characteristic of chromoblastomycosis. Morphologically, the yeast-like cells in phaeohyphomycosis appear very similar but lack internal multiseptation. Histopathologically, deep skin lesions caused by dematiaceous fungi typically exhibit pseudoepitheliomatous hyperplasia, fibrosis, and a mixed inflammatory response. This response often includes neutrophilic inflammation, chronic inflammation, granulomatous inflammation, and multinucleated giant cells. Occasionally, fragments of foreign material, such as a wooden splinter, may be observed within the lesion [12]. Recent reports indicate that *C. boppii* infections manifest as phaeohyphomycosis, with no formation of muriform cells [3,4,13].

The treatment of phaeohyphomycosis can be challenging. Commonly used antifungal agents include amphotericin B, itraconazole, voriconazole, posaconazole, caspofungin, and anidulafungin. However, the optimal duration of therapy remains unstandardized. Itraconazole is often the first-line antifungal due to its broad-spectrum activity. Despite treatment with antifungal agents, relapse is common. In localized deep cutaneous infections, systemic antifungal therapy is frequently combined with surgical excision to improve outcomes. Given that phaeohyphomycosis predominantly affects immunosuppressed patients, efforts to minimize immunosuppressive therapy to the lowest effective dose may enhance treatment success [1,12].

A joint clinical guideline published by European Society of Clinical Microbiology and Infectious Diseases (ESCMID) and European Confederation of Medical Mycology (ECMM) in 2014 provides recommendations for management. These are primarily based on expert opinion or in vitro susceptibility tests due to the lack of randomized clinical studies [14]. According to this guideline, surgical excision alone is recommended for treating localized subcutaneous phaeohyphomycosis. Additional antifungal therapy is not necessary, although it is frequently employed to ensure complete eradication in cases of incomplete excision or to prevent dissemination, particularly in immunocompromised patients [14]. *C. boppii* is currently not known to disseminate via bloodstream, in contrast to *Cladophialophora bantiana, C. modesta and C. arxii* [15].

Current case reports highlight different therapeutic approaches and outcomes for deep cutaneous *C. boppii* infections. In one case, successful treatment was achieved using a combination of local hyperthermia and systemic terbinafine (125mg/day) for 5 months [4]. Another patient received systemic itraconazole (200 mg/day) and supersaturated potassium iodide without clinical improvement. After 4 months, surgical excision of the lesion was performed, and itraconazole therapy was continued. At a 9-month follow-up, there was significant reduction in swelling, and no recurrence of the lesion was noted [3]. Beyond deep cutaneous manifestations, *C. boppii* has been implicated in a toenail and pulmonary infection. A case of toenail infection was successfully treated with oral terbinafine (250 mg/day) for 6 weeks combined with topical ciclopiroxolamine [6]. Conversely, a case involving a lung transplant recipient with a pulmonary *C. boppii* infection exemplifies the challenges of treating systemic infections. This patient initially received caspofungin and voriconazole for 3 months, but treatment failed, leading to progression of the infection despite a shift to amphotericin B [8].

In our case, the patient was successfully treated with local excision followed by a relatively short course of itraconazole (200 mg/day for three weeks). This approach contrasts with previously reported cases of deep cutaneous *C. boppii* infections, which often involved prolonged antifungal therapy with itraconazole or systemic terbinafine over several months. This outcome can likely be attributed to the presumably radical excision of the lesion.

Notably, in a prior case, itraconazole (200 mg/day) was ineffective until surgical excision was performed. This observation emphasizes the potential importance of surgical excision in reducing the fungal burden and enhancing treatment efficacy. Local heat therapy has also been reported as an alternative or adjunct treatment, though its effectiveness compared to surgical excision remains uncertain and warrants further investigation [3,4].

Our findings suggest that prolonged antifungal therapy may not be necessary to achieve favorable outcomes in localized subcutaneous *C*. *boppii* infections, aligning with the joint clinical guidelines by ESCMID and ECMM for managing phaeohyphomycosis. Following complete excision, opting for no antifungal therapy or a short course may reduce the risk of adverse effects associated with pharmacological treatment, as illustrated by our patient, who developed side effects from itraconazole.

## CRediT authorship contribution statement

**Thuy Thanh Cavens:** Writing – original draft. **Janneke de Vries:** Writing – review & editing. **Suzanne Verleisdonk-Bolhaar:** Writing – review & editing. **Jochem B. Buil:** Writing – review & editing. **Arienne van Marion:** Writing – review & editing.

## Consent

Complete written informed consent was obtained from the patient for the publication of this case report and accompanying images.

## Funding source

There are none

## Conflict of interest

There are none.
